# Supplementary figures and images for: Gut microbe Lactiplantibacillus plantarum undergoes different evolutionary trajectories between insects and mammals
Source: BMC Biol. 2022 Dec 27;20:290. doi: 10.1186/s12915-022-01477-y (PMC9795633; doi:10.1186/s12915-022-01477-y)

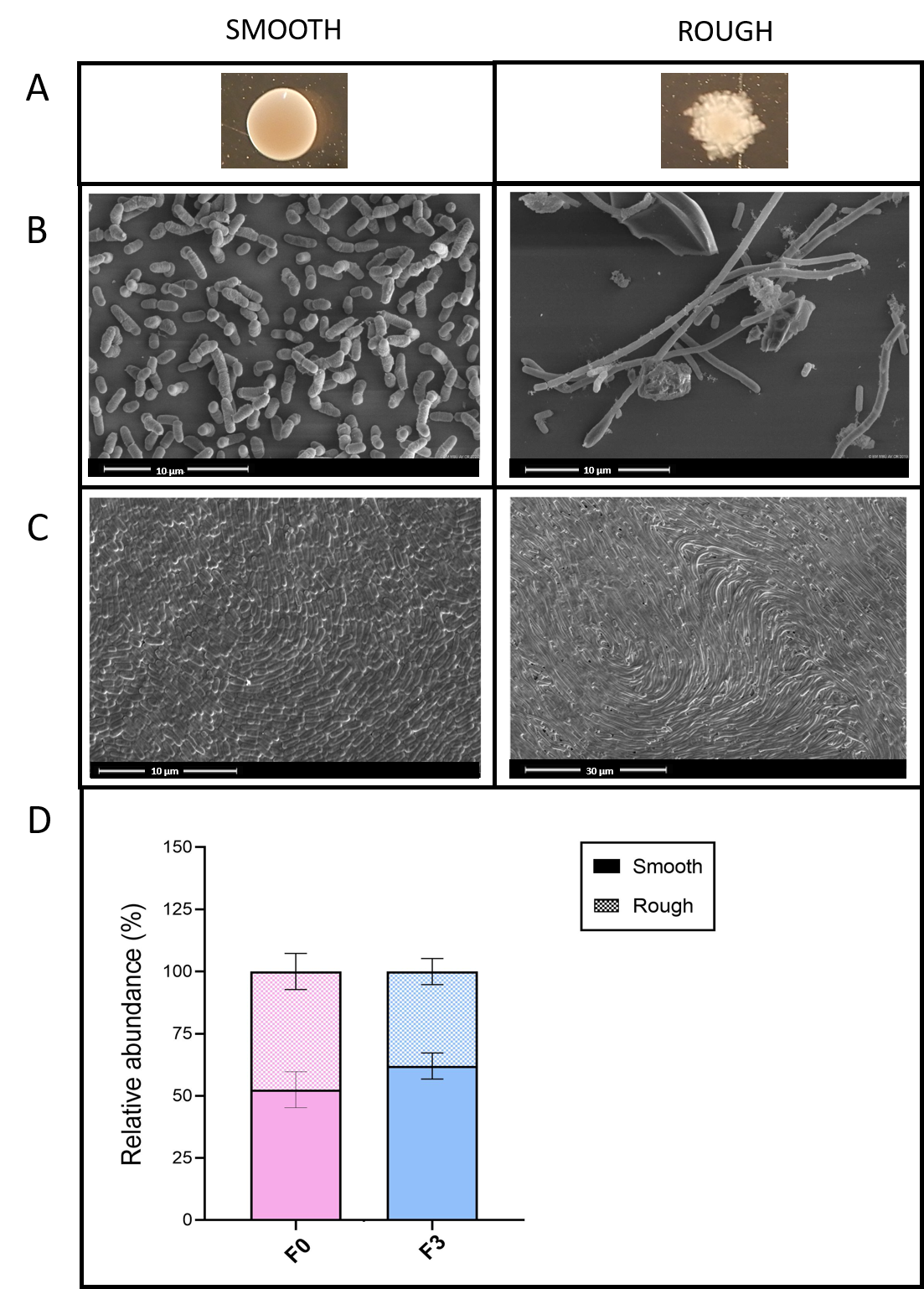

Supplement: Supplementary file 1 — Additional file 1: Figure S1. A Macroscopical appearance of smooth/rough morphotypes isolated during Lp evolution in the mouse intestine. B, C Microscopical appearance of smooth/rough Lp morphotypes at the electron microscope. d Relative abundance of smooth and rough Lp morphotypes observed at mice generations 0 (F0) and 3 (F3). Lines above each bar indicate the standard error of the mean (SEM) determined by considering three replicates for each generation. [file 12915_2022_1477_MOESM1_ESM.tif]

Population Size (total CFUs)

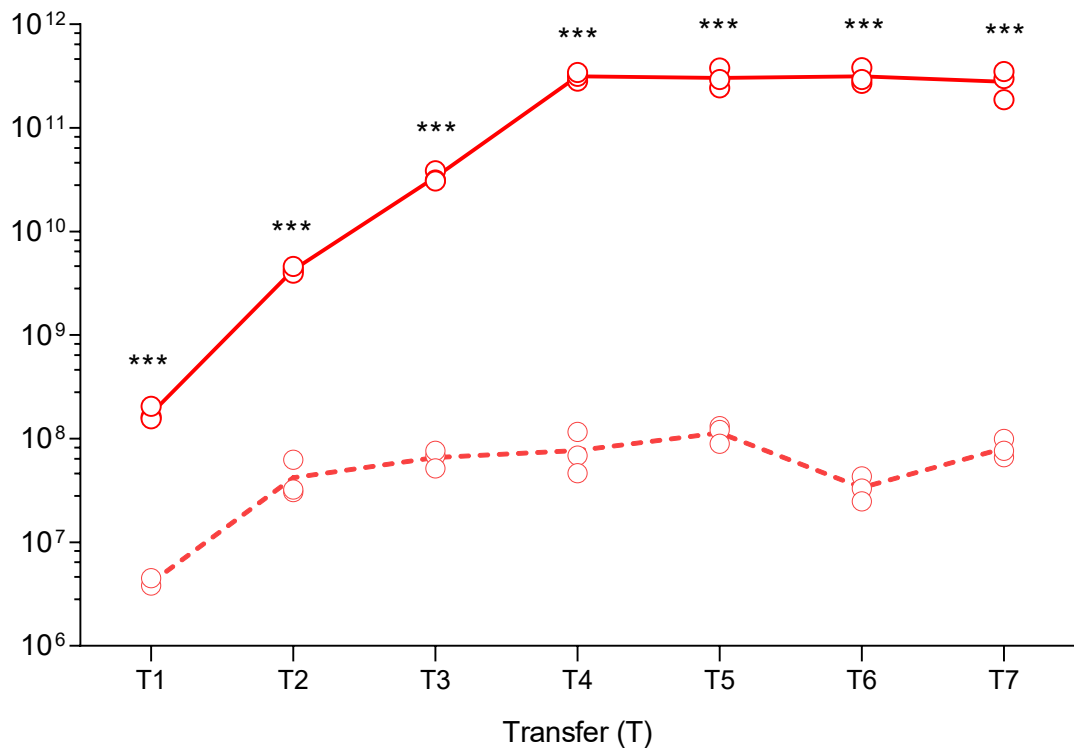

○ MRS  
○ MRS+0.3%BA

Supplement: Supplementary file 2 — Additional file 2: Figure S2. LpNIZO2877growth monitored during serial Transfers (T) in MRS broth and MRS broth added to with 0.3% bile acid (BA). At each transfer, the three circles represent the growth obtained from each of the three experimental replicates. Asterisks refer to statistical comparison between bacterial CFUs obtained from the two experimental conditions at each transfer (unpaired t-test; *** p < 0.001; df = 4). [file 12915_2022_1477_MOESM2_ESM.pdf]

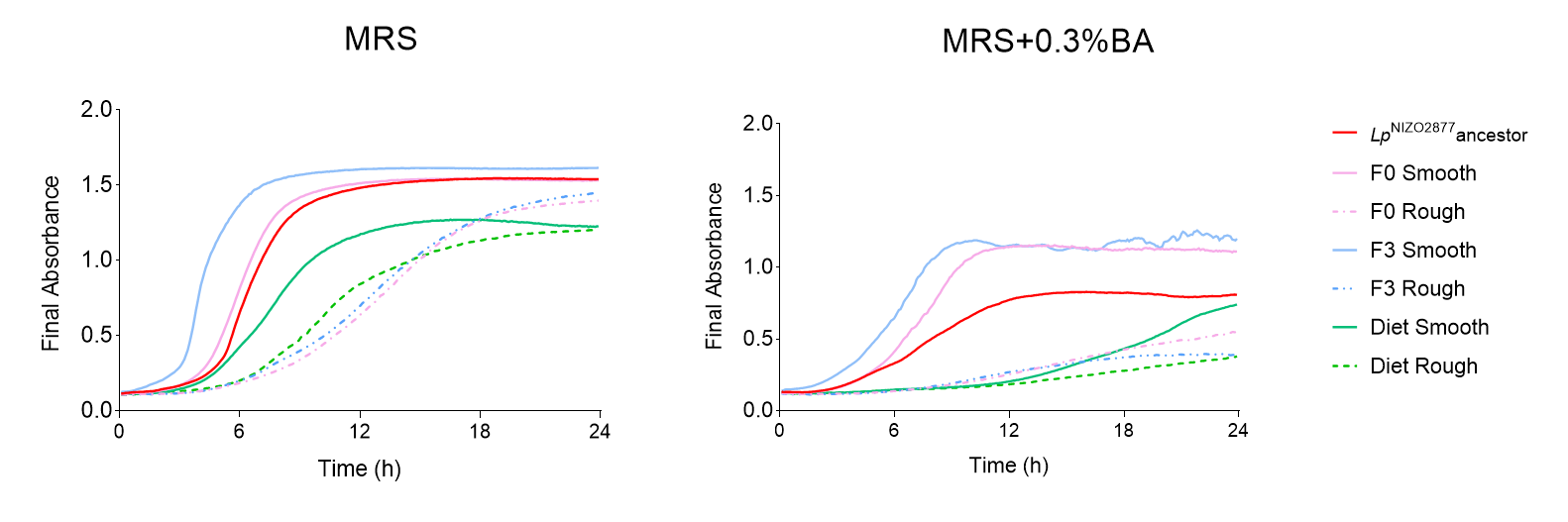

Supplement: Supplementary file 3 — Additional file 3: Figure S3. Growth curves of the Lp strains under standard growth conditions (MRS broth) and in MRS broth added to with 0.3% bile acid (BA). Each curve represents the mean of at least three replicates. [file 12915_2022_1477_MOESM3_ESM.tif]

**A** *Lp*<sup>NIZO2877</sup> - ancestor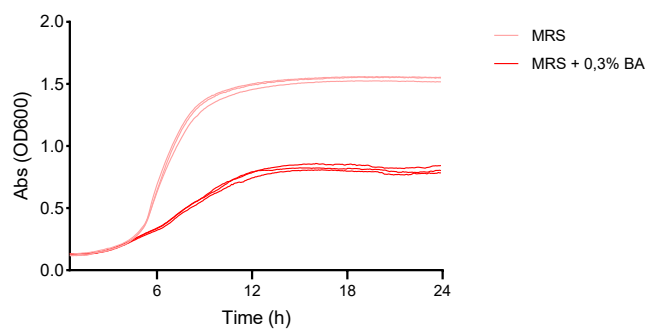**B** F0 Smooth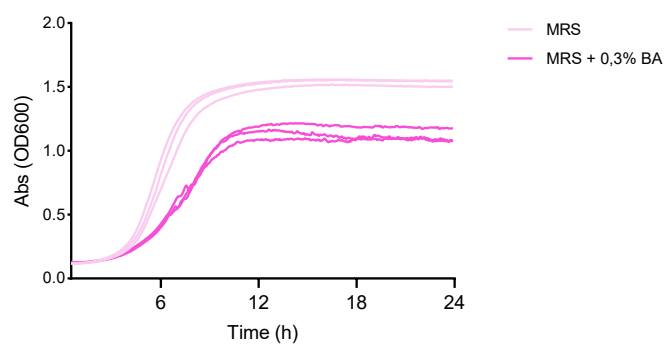**C** F0 Rough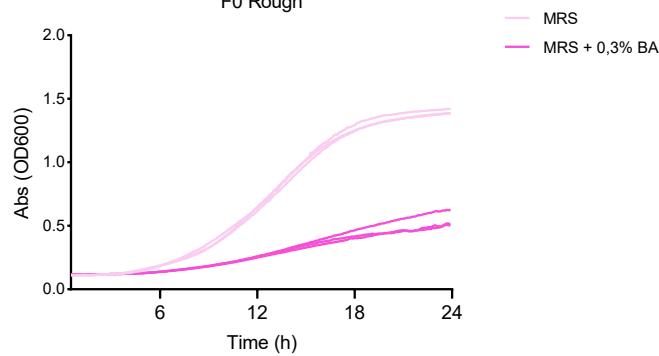**D** F3 Smooth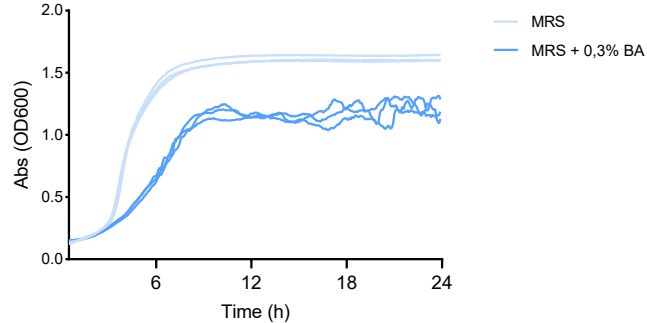**E** F3 Rough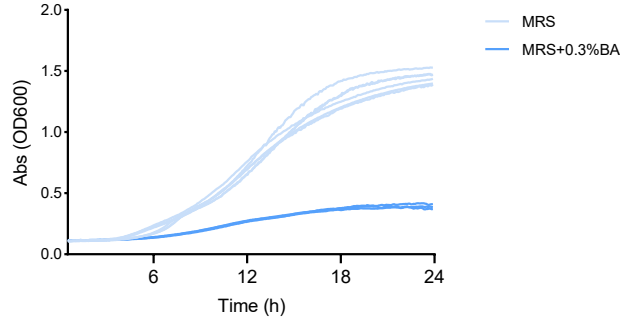**F** Diet Smooth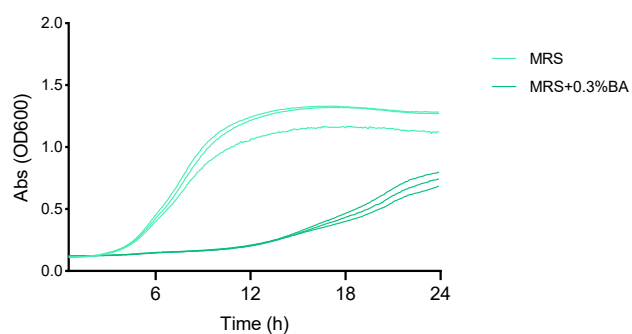**G** Diet Rough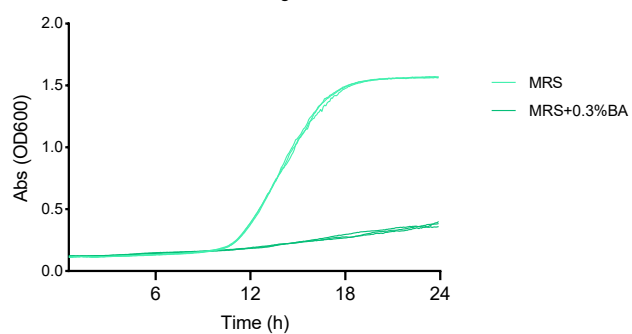

Supplement: Supplementary file 4 — Additional file 4: Figure S4. Standard growth curves of the Lp strains cultured in MRS broth and MRS broth + 0.3% bile acid (BA). The strains tested include (A) the LpNIZO2877ancestor; (B, C) Smooth and Rough colonies isolated from mice generation 0; (D, E) Smooth and rough colonies isolated from mice generation 3; (F, G) Smooth and Rough colonies isolated from the Diet setup. [file 12915_2022_1477_MOESM4_ESM.pdf]

Distance from *mutS*  $\Delta$ 1303 trajectory

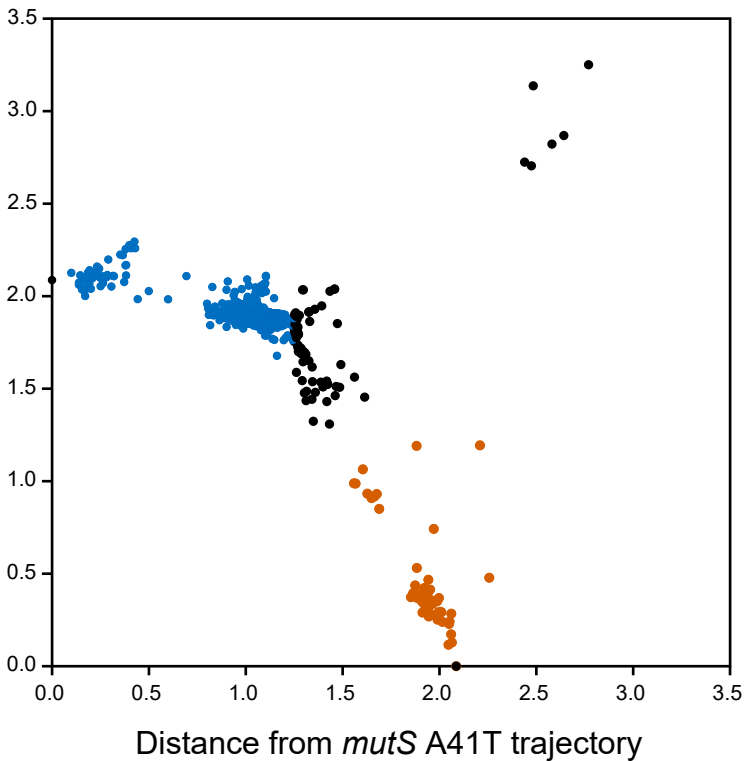

Supplement: Supplementary file 5 — Additional file 5: Figure S5. Total Manhattan distances between the frequencies of different mutations and the mutS A41T and mutS Δ1303 alleles over all samples from the mouse evolution experiment were used to classify mutations as occurring in each hypermutator lineage for examining the base substitution spectra. Mutations with distances < 0.4 to both lineages rose to a high frequency along with each mutS mutation. The cluster of mutations at a distance of ~1.0 from mutS A41T swept within this lineage later in the experiment. [file 12915_2022_1477_MOESM5_ESM.pdf]

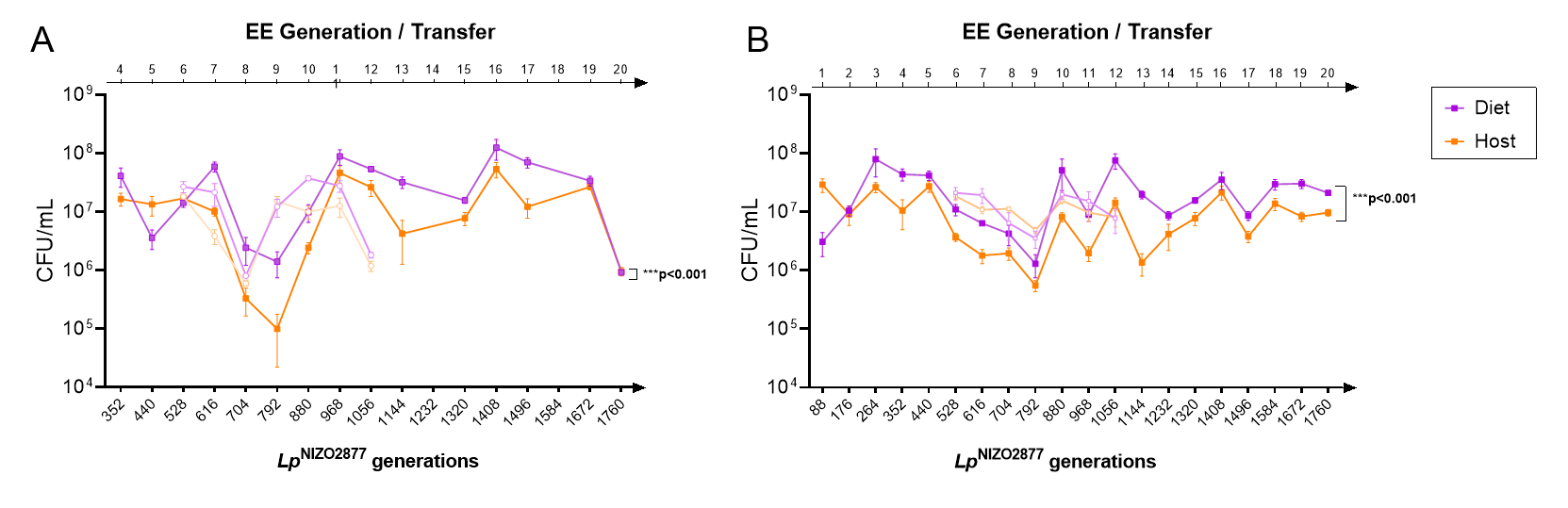

Supplement: Supplementary file 6 — Additional file 6: Figure S6. Bold full lines indicate LpNIZO2877growth monitored after 7 (A) and 11 days (B) of incubation in the presence (Host setup) or absence (Diet setup) of Drosophila. Lighter full lines indicate the re-monitoring of Lp growth from Generations/Transfers 6 to 12. ANCOVA (* p < 0.01, ** p < 0.001, and *** p < 0.0001). [file 12915_2022_1477_MOESM6_ESM.tif]

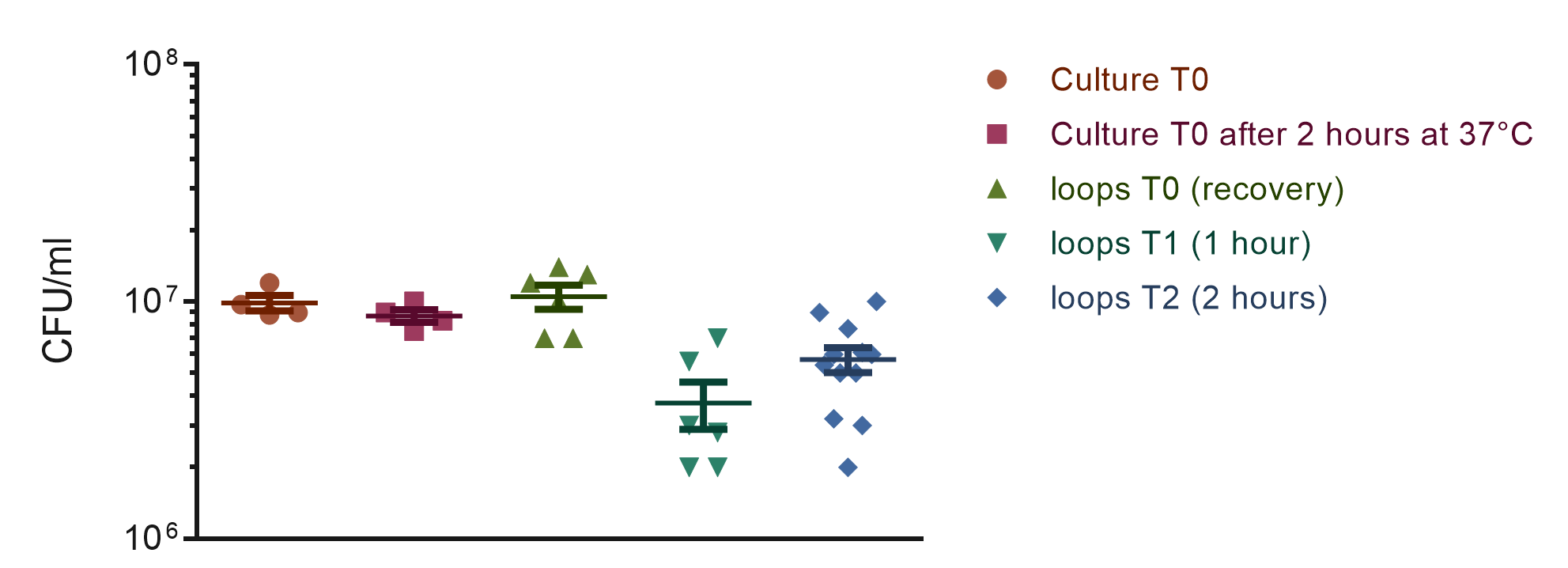

Supplement: Supplementary file 7 — Additional file 7: Figure S7. L. plantarum loads retrieved in the jejunal loops of germ-free mice. Bars indicate the standard error of the mean (SEM). [file 12915_2022_1477_MOESM7_ESM.tif]
