# Supplementary material for: Gut microbe Lactiplantibacillus plantarum undergoes different evolutionary trajectories between insects and mammals
Source: BMC Biol. 2022 Dec 27;20:290. doi: 10.1186/s12915-022-01477-y (PMC9795633; doi:10.1186/s12915-022-01477-y)
Supplement: Supplementary file 16 — Additional file 16: Table S9. Primer sequences. [file 12915_2022_1477_MOESM16_ESM.docx]

| **Primer** | **Sequence (5’-3’)** | **Annealing temp (°C)** | **Reference** |
| --- | --- | --- | --- |
| *ackA_F* | TAAGACGCAAGATACCCGTG | 62 | [87] |
| *acka_R* | ACGCACAATCATCAGCTCTT | 62 | [87] |
| *16S_UniF* | GTGSTGCAYGGYTGTCGTCA | 70 | [148] |
| *16S_UniR* | ACGTCRTCCMCACCTTCCTC | 68 | [148] |
